# Effects of Proteolytic and Lipolytic Enzyme Supplementations on Lipolysis and Proteolysis Characteristics of White Cheeses

**DOI:** 10.3390/foods7080125

**Published:** 2018-08-08

**Authors:** Oya Berkay Karaca, Mehmet Güven

**Affiliations:** 1 Karataş School of Tourism and Hotel Management, Çukurova University, Adana 01903, Turkey; 2 Department of Food Engineering, Agricultural Faculty, Çukurova University, Adana 01330, Turkey; mguven@cu.edu.tr

**Keywords:** white cheese, accelerate, lipase, protease, mineral, free amino acid, ripening

## Abstract

Effects of proteolytic (Neutrase, *Bacillus subtilis*-originate, 0.20 (P1) and 0.40 g 100 L^−1^ (P2)) and lipolytic (Piccantase A, *Mucor miehei*-originated, 0.05 (L1) and 0.10 g 100 L^−1^ (L2)) enzyme supplementations to cheese milk on lipolysis and proteolysis characteristics of 90-day ripened cheese samples were investigated in this study. While enzyme supplementation did not have significant effects on titratable acidity, fat and protease-peptone nitrogen ratios of cheese samples, dry matter, salt, protein, water soluble nitrogen, 12% trichloroacetic acid soluble nitrogen ratio (TCA-SN), 5% phosphotungstic acid soluble nitrogen (PTA-SN), casein nitrogen ratios, penetrometer value, total free fatty acids (TFFA) and total free amino acids (TFAA) were significantly influenced by enzyme supplementations. Individual free amino acids (15 of them) were also determined. Free amino acid contents of enzyme-supplemented cheeses were higher than the control cheese and the values increased in all cheese samples with the progress of ripening (*p* < 0.05). The highest amino acids in all periods of ripening were identified as glutamic acid, lysine, proline and aspartic acid. The major (Ca, P, Na, K, Mg) and minor (Zn, Fe, Cu, Mn) mineral levels of cheeses decreased with the progress of ripening and the effects of enzyme supplementations on these attributes (except for magnesium and manganese) were found to be significant (*p* < 0.01). As to conclude, enzyme supplementations increased proteolysis and lipolysis and accelerated ripening and thus reduced ripening durations. Especially the enzyme ratios in P1 and L1 cheeses were found to be suitable for reducing the ripening period in White cheese without any adverse effects.

## 1. Introduction

Textural, functional and sensory attributes of cheese usually develop with the progress of ripening. Ripening is a costly process and ripening durations largely vary based on the type of cheese [1]. It is a quite complex and slow biochemical process [2] and involves three basic reactions as of glycolysis, lipolysis and proteolysis [3]. Proteolysis is the primary reaction takes place throughout the ripening cheese [4,5] and it is the key factor to promote the diversity in texture and flavor [6]. During the primary proteolysis, residual rennet enzymes together with milk proteases hydrolyze casein and thus produce large or medium-size peptides. Then in the secondary proteolysis, proteins and large peptides are gradually hydrolyzed into smaller peptides and amino acids with the aid of intracellular and extracellular enzymes of starter cultures or other cheese microorganisms [7]. Such a breakdown of protein networks plays a significant role in textural attributes and in the release of free amino acids with a key role in production of sapid compounds [8]. Cheese flavor is directly related to release of fatty acids during the lipolysis [9]. Since ripening is a quite costly process, various economic and technical processes were practiced accelerating the process and to reduce process costs [10]. Cheese should be stored for certain durations for proper ripening. However, such a longer storage duration requires high investment costs. Shortened storage durations may increase utilization capacities of the ripening rooms, increase production capacities, reduce production costs and provide significant economic contributions to producers [11,12].

Elevated ripening temperatures, addition of enzymes (recombinant enzymes, exogenous enzymes and microencapsulation of enzymes) and starters (attenuated, adjunct and genetically modified cultures) addition of cheese slurry and high-pressure treatment are methods used to accelerate cheese ripening. Among these practices, enzyme supplementation to the milk or curd and elevated ripening temperatures are the most common methods [13,14,15]. While elevated ripening temperatures are able to be applied to limited number of cheese types, the other methods may have legal (genetically modified starters) and technical problems (excessive ripening with the use of some peptidase and proteinases) and such methods can also be quite costly (use of plasmin, attenuated starter culture and free amino acids) [16]. Commercial lipase and protease enzyme are also used separately or in combination to accelerate ripening process of cheese [17,18,19,20,21,22,23]. In this study, two previously determined different doses of *Bacillus subtilis*-originated neutral protease (Neutrase) and *Mucor miehei*-originated fungal esterase lipase (Piccantase A) enzymes were used in cheese production and control cheese was produced without any microbial enzyme supplementation. Then produced cheeses were ripened for 3 months. Experiments were conducted in 3 replications and physico-chemical characteristics, nitrogen fractions, free amino acid and mineral compositions of white cheeses were determined at different stages of the ripening process and the effects of enzymes and ripening period on these attributes were determined. 

## 2. Materials and Methods 

### 2.1. Materials

Raw milk supplied from morning milking of Livestock Facility of Agricultural Research and Implementation Center of Agricultural Faculty was used in cheese production. The Ezal MA014 (Ezal; Texel, Dange Saint Romain, France) starter supplied from Ezal Company, commercial ECOREN 200 rennet produced by Maysa Food Co. (Ümraniye, İstanbul, Turkey) were used. As lipolytic enzyme, *Mucor miehei*-originated Piccantase A GR (fungal esterase-lipase) enzyme supplied from DSM Food Specialties Co. (İzmir, Türkiye) (Turkish Distributor of French Gist-Brocades Co., Izmir, Turkey) and as proteolytic enzyme, *Bacillus subtilis*-originated Neutrase 1.5 MG enzymes supplied from Novozymes Enzyme Export Co. (Etiler, İstanbul) (Turkish Distributor of Novozymes A/S—Bagguaerd, Denmark) were used. Calcium chloride (Merck, Darmstandt, Germany) and NaCl (Merck) were also supplied from commercial suppliers.

### 2.2. Methods

#### 2.2.1. Manufacture of Experimental Turkish White Cheeses

Milk was checked for acidity and fats, passed through heat treatment at 68 ± 1 °C for 10 min, cooled down to 34 ± 1 °C and divided into 5 aliquots. These 5 sample aliquots were supplemented with 1% starter culture and 0.002% CaCl_2_ and subjected to pre-ripening for 30 min. Milk acidity levels were continuously monitored throughout the pre-ripening period and enzyme supplementation and fermentation was performed when the pH level reached to 6.4. Following pre-ripening, two aliquots were supplemented respectively with 0.05 g 100 L^−1^ (P1 cheese) and 0.10 g 100 L^−1^ (P2 cheese) *Bacillus subtilis*-originated neutral protease, the other two aliquots were supplemented with 0.20 g 100 L^−1^ (L1 cheese) and 0.40 g 100 L^−1^ (L2 cheese) *Mucor miehei*-originated fungal esterase lipase enzymes. The remaining last aliquot was used as control (C cheese) without any enzyme supplementation. Milk was coagulated with rennet at 32 ± 1 °C for 90 min. The coagulum was cut into 2–3 cm^3^ pieces, left to drain for 30 min. The gradually increasing weights (0.01–0.03 kg cm^−2^) were placed over the samples to press and separate whey. Then, cheese curds were cut into 7.5–8 cm^3^ pieces and salted at 12% brine and placed into 1 kg cheese containers. The containers were fully filled with 12% fresh brine, tightly closed and left for ripening in a cold storage at 7 ± 1 °C for 90 days. 

#### 2.2.2. Methods of Analysis

##### Gross Composition of Milk, Whey and Cheese Samples

Dry matter (DM) ratio of the milk and whey was determined by gravimetric method [24], fat ratio with Gerber method [25], protein ratios with Micro-Kjeldahl method [26], pH values with Inolop WTW (Weilheim, Germany) digital pH meter, titration acidity with alkaline titration [25], lactose ratios with the Layne–Eynon method [27]. Cheese yield performance was calculated as the amount of cheese (kg) produced from 100 kg milk. Cheese dry matter ratios were determined with gravimetric method [24], fat ratios with Van-Gulik butyrometer [28], protein ratios with Micro-Kjeldahl method [26], salt ratios with Mohr titration method and titration acidity with alkaline titration method [29]. Penetrometer value was determined at 4 ± 1 °C with Sur-Berlin PNR 6-brand penetrometer and the results were achieved as the penetration depth of 95.5 g penetrating tip in 5 s (1/10 mm). 

Water soluble nitrogen (WSN) ratio [30], 5% phosphotungstic acid soluble nitrogen (PTA-SN) ratio [31] and 12% trichloroacetic acid soluble nitrogen ratio (TCA-SN) [32] were determined with the Micro–Kjeldahl method [26]. The degree of ripening in terms of 12% TCA soluble nitrogen was calculated as the ratio of 12% TCA soluble nitrogen to total nitrogen (TN). Casein nitrogen ratio was determined by subtracting water soluble nitrogen ratio from total nitrogenous substance ratio and results were expressed in % nitrogen. Protease-peptone nitrogen ratio was calculated by subtracting 12% TCA soluble nitrogen ratio from water soluble nitrogen ratio [33]. 

##### Total Free Amino Acid Ratios 

Total free amino acid ratios of proteolytic enzyme-supplemented cheeses were determined spectrophotometrically in accordance with Cd-ninhidrine method [34]. Calculations were made based on created standard curve and results were expressed in mg Leu g^−1^ cheese. 

##### Total Free Fatty Acid Ratios 

Fat extractions from lipolytic enzyme-supplemented cheeses were performed in accordance with Nunez et al. [35] with slight modifications and results were expressed in terms of % oleic acid.

##### Free Amino Acid Composition

Phenomenex EZ Faast GC-FID Hydrolyzed Amino Acid Analysis by GC-FID kit (Phenomenex, Lane Cove West, NSW, Australia) was used to determine individual amino acids [36]. The procedure is briefly composed of three phases as of solid-phase extraction clean-up, derivation of the amino acids and liquid/liquid extraction phases. Zebron ZB-AAA chromatography column (10 m × 0.25 mm) fitted to a Varian CP-3800 gas chromatograph was used to analyze resultant derivative amino acids with an organic layer. The gas chromatograph was equipped with an auto-sampler and a flame ionization detector (FID 320 C, California, CA, USA) and operated at constant pressure flow mode (helium, 60 kPa, 1.5 mL/min) with a split injection ratio of 1:15. The injector temperature was 250 °C and detector temperatures was 300 °C. The column oven temperature was gradually increased from 110 °C to 310 °C with an increment ratio of 30 °C min^−1^. Then the temperature was held constant at 310 °C for 18 s. The overall run time was 7.56 min. The chromatographic data were managed by Varian Star Chromatography software (version 5.52; Varian Inc., Palo Alto, CA, USA). Amino acid quantities of proteolytic enzyme-supplemented cheese samples were calculated from the GC peaks and results were expressed in mg 100 g^−1^. These analyses were performed at the TUBITAK (The Scientific and Technical Research Council of Turkey) Food Institute of Marmara Research Centre.

##### Mineral Analyses with Atomic Absorption Spectrophotometer 

Dry-ashing method was employed in mineral analyses [37]. Phosphorus analyses of cheese samples were performed colorimetrically in a spectrophotometer (Hitachi U-2000, Minato-ku, Tokyo, Japan). Readings were performed with the standard samples prepared identical with the samples prepared in accordance with Barton (0–100 mg P mL^−1^) at 430 nm. K, Na, Ca, Mg, Fe, Cu, Zn and Mn contents were determined with the aid of an Atomic Absorption Spectrophotometer (AAS) (Varian SpectrAA220, Victoria, Australia) provided that required dilutions were performed. Resultant values were multiplied by dilution factor and expressed in mg 100 g^−1^ DM. 

##### Statistical Analyses

Statistical analyses were performed in accordance with one way ANOVA “Coincidental Parsellity Trial Plan” to determine the effects of different enzymes and ripening period and significant differences were compared with Duncan’s multiple range test. 

## 3. Results and Discussion

### 3.1. Raw Milk and Whey Characteristics and Cheese Yield Performance 

As can be seen in Table 1, average dry matter content of the raw milk was 11.23%, fat content was 3.2%, protein content was 3.13%, lactose content was 4.37%, pH was 6.61 and acidity was 6.69 °SH. Whey pH values varied between 5.62–5.69%, titration acidity values varied between 8.26–8.75 °SH, dry matter ratios between 6.52–6.64%, protein contents between 0.90–0.91%, lactose contents between 4.25–4.49% and the values were close to each other (*p* > 0.05). Fat contents of whey samples varied between 0.25–0.30% with the lowest values from the control and L1 cheese (*p* < 0.01).

Cheese yield performances varied between 12.97–14.02% (Table 2) and enzyme-supplemented cheeses had significantly different and lower values than the control cheese (*p* < 0.05). The actual performance is indicated as transition ratio of milk dry matter components into the cheese. In case of performance calculations as to have 40% dry matter ratio in white cheese, it was observed that protease and lipase enzyme-supplemented cheeses had close performance values to each other but had significantly lower values than the control cheese and decreasing performance values were observed with increasing enzyme supplementation rates (*p* < 0.05). Previous researchers also indicated that enzyme supplementation to cheese to accelerate ripening reduced performance values and such a case primarily resulted from increasing nitrogen losses in whey [14,19,38,39].

### 3.2. Gross Composition of Cheeses

Titration acidity, penetrometer values and general composition characteristics of white cheeses throughout 90-day ripening period are provided in Table 3. Titration acidity (TA) values of cheeses throughout the ripening period were close to each other, while the differences between the cheeses and enzyme dose x ripening period interaction were not significant (*p* > 0.05), but the effects of ripening period were found to be significant (*p* < 0.01). It was also reported in previous studies that enzyme-supplemented cheeses did not have significantly different acidity values from the control cheese [38,40,41]. 

Enzyme supplementation had significant effects on dry matter and protein contents of the chesses at 30th and 90th day of ripening (*p* < 0.05), ripening period and enzyme × ripening period interaction had significant effects on these attributes at *p* < 0.01 level. It was reported in previous studies that proteolytic and lipolytic enzymes did not have significant effect on dry matter content of cheeses [42,43,44,45]. Increasing acidity levels were reported to reduce water holding capacity of casein, thus increased dry matter content of the cheeses [46]. At the 90th day of ripening, protease enzyme-supplemented cheeses had significantly lower protein contents than the control cheese (*p* < 0.01) such a case was resulted from excessive protease contents. Protein contents of the cheeses had generally decreasing trends after the 30th day of ripening (*p* < 0.01). 

The effects of enzyme treatments and enzyme × ripening period interaction on fat contents of the cheeses were not found to be significant (*p* > 0.05). It was reported that lipase enzyme supplementation did not have significant effects on fat contents of Ras cheese produced with Piccantase A [42,43], on fat contents of again Domiate cheese produced by using Palatase A, Palatase M and Piccantase [46] and on fat contents of Kasher cheese produced with Palatase M 200 L [47] and increasing fat contents were reported with the progress of ripening just based on increasing dry matter contents [41]. Slightly lower fat contents of lipase enzyme-supplemented cheeses than the others on 90th day of ripening can be attributed to high lipase enzyme activity and resultant release of fat from the cheeses. High fat contents of protease enzyme-supplemented chesses than the others can be attributed to release of nitrogenous by-products from the cheeses because of proteolysis with the progress of ripening and resultant decreasing protein content and relatively increasing fat and other compound content of dry matter. Present analyses revealed that enzyme treatments had significant effects on salt ratios throughput the ripening period (*p* < 0.05), except for 90th day of ripening. On the other hand, ripening period and enzyme × ripening period interaction had significant effects on the salt ratios at *p* < 0.01 level. The fluctuations in dry matter salt ratios of lipase-supplemented cheeses can be attributed to changes in dry matter contents of these cheeses. 

As compared to the 1st day of ripening, cheeses hardened on 15th day of ripening, but softened from the 30th day until the end of ripening period. Enzyme treatments, enzyme x ripening period interaction had significant effects on penetrometer value (PV) (*p* < 0.01). As compared to the control cheese, at the last period of ripening, proteolytic enzyme-supplemented cheeses had either similar or harder texture and lipase enzyme-supplemented cheeses had softer texture (*p* < 0.01). Such a case can be attributed to high lipase levels of the cheeses [40,48]. 

### 3.3. Nitrogen Fractions and Ripening Index of the Cheeses 

In terms of water soluble nitrogen and 12% TCA soluble nitrogen contents (Table 4) of the cheeses, it was observed that protease enzyme-supplemented cheeses were significantly different from the others on the last day of ripening and significant increases were observed throughout the ripening period (*p* < 0.01). Nunez et al. [48]. reported that water soluble nitrogen formation in cheeses was more influenced from Neutrase L and Novozym proteinase enzyme supplementations than the 12% TCA-SN and 5% PTA-SN soluble nitrogen formation. Nasr [38] for Piccantase enzyme-supplemented Romi cheeses, Kheadr et al. [49] for *Mucor miehei*-originated Palatase M and *Aspergillus niger*-originated Lipase 50 enzyme-supplemented Cheddar cheeses, reported that water soluble nitrogen and non-protein nitrogenous substance contents were slightly higher than the control cheese and these values increased with the progress of ripening.

Peptide and amino acids with a molecular weight less than 600 dalton are significant indicators of cheese aroma levels [18] and these substances dissolved in 5% PTA-SN [50]. The differences in peptide and amino acid quantities dissolved in 5% PTA-SN at different ripening periods were found to be significant, proteolytic enzyme quantities did not yield significant differences (*p* > 0.05) and protease enzyme-supplemented cheeses were significantly different from lipase enzyme-supplemented chesses (*p* < 0.01). Such a case can be attributed to enhanced peptidase activity arising from the higher levels of primary proteolysis. Researchers indicated the primary reason for such cases as solubility of small molecule peptides and amino acids generated throughout the ripening duration in 5% PTA-SN. Increasing free amino acid quantities of these cheeses also support such an idea. 

With regard to casein ratios in % nitrogen contents, it was observed that enzyme treatments (except for 30th day of ripening), ripening period, enzyme × ripening period interaction had significant effects on casein % nitrogen values, casein % nitrogen values decreased throughout the ripening duration (*p* < 0.01) and such a decrease was higher in proteolytic enzyme-supplemented cheeses than the control cheese (*p* < 0.01). Decreasing casein nitrogen quantities can be attributed to hydrolysis of α- and β-casein into peptides and amino acids. 

Enzyme treatments, enzyme × ripening period interaction had significant effects on protease-peptone nitrogen values at *p* < 0.05 levels and ripening period had significant effects at *p* < 0.01 level. Except for 30th day of ripening, protease-peptone nitrogen rations of cheeses increased throughout the ripening period, except for 30th day of ripening (*p* < 0.01). Karaca and Guven [51] indicated regular decreases in casein nitrogen and increases in protease-peptone nitrogen ratios of white cheeses during the ripening process and reported the highest change in cheeses including *Mucor miehei*-originated protease enzyme.

**Figure 1:**
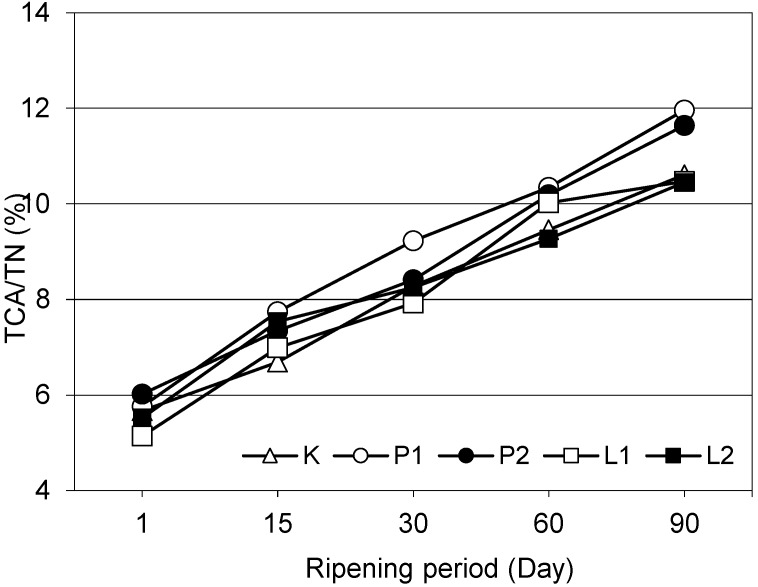
Ripening indices trichloroacetic acid soluble nitrogen (TCA-SN/TN) of White cheeses during ripening.

Except for the 15th and 30th day of ripening, lipolytic and proteolytic enzyme-supplementations had significant effects on 12% TCA soluble nitrogen ratios, in other words on ripening index (Figure 1), of the cheeses (*p* < 0.01). On the 90th day of ripening, lipase enzyme-supplemented L1 and L2 chesses had similar 12% TCA soluble nitrogen ratios with the control cheese and such a ratio of proteolytic enzyme-supplemented P1 and P2 cheeses were significantly higher than the other cheeses (*p* < 0.01). On the other hand, enzyme quantities did not have significant effects on this ratio (*p* > 0.05). The 12% TCA soluble nitrogen ratios of white cheeses regularly increased with the progress of ripening (*p* < 0.01). Significant increases in 12% TCA soluble nitrogen ratios were also reported with the progress of ripening in Feta cheese by Valsamaki et al. [52], in Manchego cheese by Gaya et al. [53] and in white cheeses by Güven and Karaca [54], Cinbaş and Kılıç [55]. Significantly higher TCA soluble nitrogen ratios than the control cheese and increasing values with the progress of ripening were also reported for *Bacillus subtilis*-originated neutral protease-supplemented Manchego cheese [48,56] and Cheddar cheese [57].

### 3.4. Total Free Amino Acids 

**Figure 2:**
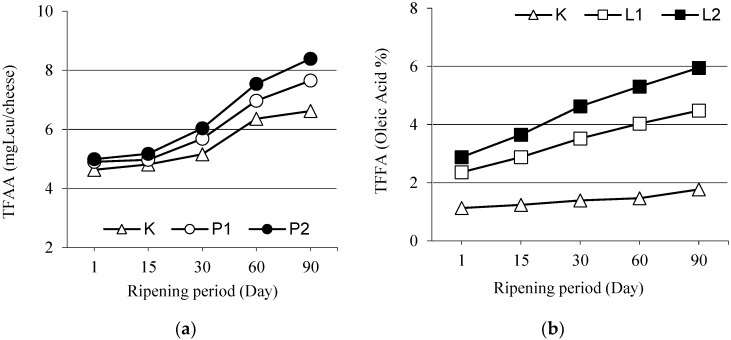
(**a**) Total free amino acids (TFAA); and (**b**) Total free fatty acids (TFFA) contents of White cheeses during ripening.

Total free amino acid contents of the white cheeses are presented as means ± standard errors in Figure 2. Enzyme treatments, ripening period and enzyme × ripening period interaction had significant effects on total free amino acid contents of the cheeses. Total free amino acid contents of protease-supplemented cheeses were significantly higher than the control cheese on the 1st day of ripening (*p* < 0.01) and such a case went on throughout the ripening duration (*p* < 0.01). 

Total free amino acid contents of the cheeses increased with increasing enzyme quantities and total free amino acid content of P2 cheese was significantly higher than the P1 cheese (*p* < 0.01). Faster increases were also reported in total free amino acid levels of protease enzyme-supplemented cheeses throughout the ripening process [19,58,59]. Increasing total free amino acid contents with the progress of ripening were also reported for Feta cheese [52,60] and for Ras cheese [61]. 

### 3.5. Individual Free Amino Acid Composition

Individual free amino acid composition of cheeses (15 amino acids) is provided in Table 5. 

Free amino acid content of cheese is largely depending on manufacturing technology and ripening period. However, the primary factors responsible for amino acid release were indicated as non-starter lactic acid bacteria and proteolytic enzymes in the cheese [4]. They contribute to the overall characteristic flavor of different cheese varieties [6] and describe how far the ripening has proceeded [62]. As compared to the control cheese, protease enzyme-supplemented cheeses had significantly higher proline, aspartate and glutamate amino acid concentrations on the 30th day of ripening (*p* < 0.05). On the other hand, on the 90th day of ripening, proline, aspartate, glutamate and histidine amino acid concentrations of enzyme-supplemented cheeses were different from each other (*p* < 0.05). A general increase was observed in free amino acid concentrations of the control and protease enzyme-supplemented cheeses throughout the ripening period. The proteolytic activity during cheese ripening produces a higher content of short-chain peptides and FAAs [63]. The increase in aspartate and methionine concentrations of especially the proteolytic enzyme-supplemented cheeses throughout the ripening period was quite higher than the control treatment. In all periods of ripening of white cheeses, the highest amino acids were identified as glutamate, leucine, proline and aspartate and these amino acids increased with increasing protease enzyme quantities (*p* < 0.05).

### 3.6. Total Free Fatty Acids (TFFA) 

Total free fatty acid quantities of lipase enzyme-supplemented cheeses (Figure 2) were higher than the control cheese in all ripening periods and total fatty acid quantities increased with increasing enzyme quantities (*p* < 0.01). Total free fatty acids of all cheeses regularly increased with the progress of ripening and enzyme x ripening period interaction had significant effects on these increases in all periods of ripening (*p* < 0.01). Previous researchers also reported increasing total fatty acid quantities throughout the ripening process in different cheese types [41,61]. Dinkçi [64] and Koçak et al. [45] indicated significant effects of lipase enzyme (Piccantase A, Palatase M 200 L) concentrations on total fatty acid quantities of cheese samples and reported increasing total fatty acid quantities with increasing enzyme concentrations. 

### 3.7. Mineral Composition 

Some minerals move from the inside out with the effect of pH and thus variations are observed in mineral concentrations of the final products [65]. On 30th day of ripening, calcium and magnesium contents of the cheese samples varied respectively between 687–764 and between 204–211 mg 100 g^−1^ (Table 6). 

The values decreased on 90th day and varied respectively between 631–718 mg 100 g^−1^ and between 175–189 mg 100 g^−1^. The control and lipase enzyme-supplemented cheeses had higher calcium, sodium and phosphorus contents than the protease enzyme-supplemented cheeses. Statistical analyses revealed that enzyme treatments had significant effects on calcium, sodium, magnesium, phosphorus and zinc contents of while cheeses (*p* < 0.01) but did not have significant effects on potassium contents (*p* > 0.05). Ripening period and enzyme × ripening period interaction had significant effects on all minerals (*p* < 0.05). On the 30th day of ripening, the highest zinc (Zn) content (4.63 mg 100 g^−1^) was obtained from the control cheese and it was respectively followed by P2, L2, L1 and P1 cheeses (*p* < 0.05). The differences in iron (Fe) contents of the cheeses were not found to be significant on 30th day of ripening (*p* > 0.05). Enzyme treatments did not have significant effects on iron and manganese contents (*p* > 0.05) but had significant effects on copper contents (*p* < 0.01). Effects of ripening period and enzyme × ripening period interaction were not also found to be significant (*p* > 0.05). Major and minor minerals of cheese samples decreased with the progress of ripening. Such decreases throughout the ripening process were attributed to mineral passage into brine in time.

## 4. Conclusions

In this study, cheese was produced with Bacillus subtilis-originated neutral protease (Neutrase) and Mucor miehei-originated fungal esterase lipase (Piccantase A) enzyme supplementation at two previously determined quantities. The control cheese was produced without any microbial enzyme supplementations. Cheese samples were ripened for 3 months. Lipase enzyme supplementation increased whey fat content. In case of performance calculations as to have 40% dry matter ratio in white cheese, it was observed that enzyme-supplemented cheeses (except for P1 cheese) had significantly lower cheese yield performance than the control cheese. While titration acidity, dry matter content, water soluble nitrogen, 12% TCA soluble nitrogen, 5% PTA soluble nitrogen, protease-peptone nitrogen, penetrometer value, total free fat and amino acid concentrations increased, salt, casein nitrogen and mineral concentrations decreased with the progress of ripening. The highest major minerals were identified as sodium, calcium and phosphorus and the minor minerals as zinc and iron, respectively. The highest penetrometer values, in other words the softest texture, were obtained from lipase enzyme-supplemented chesses. It was concluded based on present findings that enzyme supplementations increased secondary proteolysis especially increasing free amino acids contents and lipolysis and accelerated ripening and thus reduced ripening period. The use of 0.20 g 100 L^−1^ Neutrase and 0.05 g 100 L^−1^ Piccantase A in White cheeses is recommended for providing significant economic contributions (increasing the capacity of ripening rooms, reducing costs and time) to producers. It has been determined that the use of proteolytic and lipolytic enzymes at higher levels causes a hard and brittle structure in cheeses and a bitter taste due to excessive proteolysis and lipolysis. 

## Figures and Tables

**Table 1:** Composition of the raw cow milk and whey of White cheeses (*n* = 3).

Properties	Raw Cow Milk	Whey				
		C *	P1	P2	L1	L2
pH	6.61 ± 0.01	5.63 ± 0.02 ^a^	5.62 ± 0.03 ^a^	5.62 ± 0.02 ^a^	5.67 ± 0.10 ^a^	5.69 ± 0.10 ^a^
TA **	6.69 ± 0.02	8.74 ± 0.12 ^a^	8.71 ± 0.08 ^a^	8.75 ± 0.09 ^a^	8.32 ± 0.81 ^a^	8.26 ± 0.67 ^a^
DM ** (%)	11.23 ± 0.10	6.52 ± 0.08 ^a^	6.55 ± 0.09 ^a^	6.64 ± 0.08 ^a^	6.56 ± 0.10 ^a^	6.64 ± 0.08 ^a^
Fat (%)	3.20 ± 0.00	0.25 ± 0.03 ^b^	0.30 ± 0.00 ^a^	0.30 ± 0.00 ^a^	0.26 ± 0.05 ^b^	0.30 ± 0.00 ^a^
Protein (%)	3.13 ± 0.02	0.91 ± 0.01 ^a^	0.91 ± 0.02 ^a^	0.90 ± 0.02 ^a^	0.91 ± 0.01 ^a^	0.91 ± 0.01 ^a^
Lactose (%)	4.37 ± 0.06	4.27 ± 0.21 ^a^	4.34 ± 0.28 ^a^	4.49 ± 0.14 ^a^	4.25 ± 0.13 ^a^	4.32 ± 0.10 ^a^

^a,b^ Means in the same row followed by different letters were significantly different (*p* < 0.05). * Cheeses: C: Control without enzymes; P1; 0.05 g 100 L^−1^ protease, *Bacillus subtilis*; P2: 0.10 g 100 L^−1^ protease, *Bacillus subtilis*; L1: 0.20 g 100 L^−1^ lipase, *Mucor miehei*; L2: 0.40 g 100 L^−1^ lipase, *Mucor miehei*. ** DM: Dry Matter; TA: Titratable acidity as °SH, Soxhelet Henkel Unit.

**Table 2:** Yield rates of White cheeses (*n* = 3).

	C *	P1	P2	L1	L2
Yield (%)	14.02 ± 0.31 ^a^	13.26 ± 0.32 ^b^	12.97 ± 0.22 ^b^	13.24 ± 0.63 ^b^	12.99 ± 0.42 ^b^
Yield (%40)	15.53 ± 0.36 ^a^	14.79 ± 0.14 ^ab^	14.20 ± 0.21 ^b^	14.49 ± 0.68 ^b^	14.33 ± 0.45 ^b^

^a,b^ Means in the same row followed by different letters were significantly different (*p* < 0.05). * Cheeses: C: Control without enzymes; P1; 0.05 g 100 L^−1^ protease, *Bacillus subtilis*; P2: 0.10 g 100 L^−1^ protease, *Bacillus subtilis*; L1: 0.20 g 100 L^−1^ lipase, *Mucor miehei*; L2: 0.40 g 100 L^−1^ lipase, *Mucor miehei*.

**Table 3:** Titratable acidity, penetrometer values and mean composition of experimental White cheeses during ripening (*n* = 3).

	Day	1	30	60	90
**Dry matter (%)**	C *	44.28 ± 0.101C ^a^	45.43 ± 0.498B ^b^	45.94 ± 0.635B ^a^	46.93 ± 0.257A ^a^
	**P1**	44.63 ± 0.666B ^a^	46.27 ± 0.342A ^ab^	46.75 ± 0.723A ^a^	47.19 ± 0.732A ^a^
	**P2**	43.80 ± 0.810B ^a^	46.90 ± 0.580A ^a^	46.49 ± 0.897A ^a^	47.48 ± 0.622A ^a^
	**L1**	43.76 ± 0.042C ^a^	45.42 ± 0.605B ^b^	47.21 ± 1.257A ^a^	45.62 ± 0.222B ^b^
	**L2**	44.12 ± 0.055B ^a^	46.69 ± 0.298A ^a^	46.26 ± 0.573A ^a^	46.71 ± 0.361A ^a^
**Fat (%)**	**C**	22.00 ± 0.000C ^a^	22.92 ± 0.144BC ^a^	23.75 ± 0.661AB ^a^	24.08 ± 0.144A ^a^
	**P1**	22.58 ± 0.520B ^a^	23.33 ± 0.382B ^a^	24.58 ± 0.804A ^a^	24.67 ± 0.577A ^a^
	**P2**	21.50 ± 0.500C^a^	23.42 ± 0.382B ^a^	24.08 ± 0.629AB ^a^	24.83 ± 0.520A ^a^
	**L1**	21.83 ± 0.289B ^a^	22.83 ± 0.289AB ^a^	23.83 ± 0.764A ^a^	23.42 ± 0.382A ^a^
	**L2**	22.00 ± 0.000B ^a^	23.08 ± 0.144A ^a^	23.25 ± 0.250A ^a^	23.58 ± 0.382A ^a^
**Protein (%)**	**C**	17.39 ± 0.280A ^a^	18.11 ± 0.604A ^b^	18.11 ± 0.436A ^a^	18.39 ± 0.111A ^ab^
	**P1**	17.32 ± 0.346B ^a^	18.35 ± 0.066A ^b^	18.19 ± 0.086A ^a^	18.11 ± 0.053A ^bc^
	**P2**	17.38 ± 0.108C ^a^	19.37 ± 0.217A ^a^	18.31 ± 0.065B ^a^	17.71 ± 0.456C ^c^
	**L1**	17.53 ± 0.260B ^a^	18.26 ± 0.120B ^b^	19.06 ± 0.702A ^a^	17.88 ± 0.188B ^bc^
	**L2**	17.42 ± 0.163C ^a^	19.23 ± 0.423A ^a^	18.34 ± 0.038B ^a^	18.70 ± 0.401AB ^a^
**Salt (%)**	**C**	2.24 ± 0.025A ^b^	2.13 ± 0.066AB ^a^	1.87 ± 0.179BC ^ab^	1.67 ± 0.299C ^a^
	**P1**	2.15 ± 0.010A ^c^	1.94 ± 0.017AB ^c^	1.69 ± 0.144B ^b^	1.89 ± 0.221B ^a^
	**P2**	2.53 ± 0.047A ^a^	2.09 ± 0.100B ^ab^	1.65 ± 0.112C ^b^	1.96 ± 0.291B ^a^
	**L1**	1.79 ± 0.068BC ^e^	2.08 ± 0.012A ^ab^	1.74 ± 0.105C ^b^	1.95 ± 0.166AB ^a^
	**L2**	1.93 ± 0.050A ^d^	2.02 ± 0.032A ^bc^	2.08 ± 0.085A ^a^	2.06 ± 0.125A ^a^
PV **	**C**	68.83 ± 0.289A ^a^	58.00 ± 5.196BC ^a^	61.67 ± 0.577B ^a^	63.00 ± 0.500AB ^c^
	**P1**	55.50 ± 0.866C ^d^	53.17 ± 0.289D ^bc^	58.67 ± 0.289B ^b^	63.83 ± 0.289A ^c^
	**P2**	58.83 ± 1.756B ^c^	49.17 ± 0.764C ^c^	58.17 ± 0.577B ^b^	68.33 ± 0.577A ^b^
	**L1**	60.00 ± 0.000B ^c^	54.00 ± 0.866D ^ab^	58.33 ± 1.527C ^b^	70.17 ± 0.289A ^a^
	**L2**	63.17 ± 0.289B ^b^	50.67 ± 0.764D ^bc^	59.33 ± 0.764C ^b^	70.17 ± 0.577A ^a^
TA ** (LA%)	**C**	2.02 ± 0.035C ^a^	2.26 ± 0.040B ^a^	2.38 ± 0.097AB ^a^	2.51 ± 0.076A ^a^
	**P1**	2.06 ± 0.104C ^a^	2.23 ± 0.040BC ^a^	2.39 ± 0.147AB ^a^	2.52 ± 0.102A ^a^
	**P2**	2.02 ± 0.015C ^a^	2.24 ± 0.031B ^a^	2.28 ± 0.065B ^a^	2.41 ± 0.000A ^a^
	**L1**	2.03 ± 0.064D ^a^	2.30 ± 0.096BC ^a^	2.44 ± 0.006AB ^a^	2.47 ± 0.127A ^a^
	**L2**	2.05 ± 0.146B ^a^	2.34 ± 0.038A ^a^	2.40 ± 0.029A ^a^	2.46 ± 0.031A ^a^

A,B,C,D Means in the same row followed by different letters were significantly different (*p* < 0.05); ^a,b,c,d,e^ Means in the same column followed by different letters significantly different (*p* < 0.05); * Cheeses: C: Control without enzymes; P1; 0.05 g 100 L^−1^ protease, *Bacillus subtilis*; P2: 0.10 g 100 L^−1^ protease, *Bacillus subtilis*; L1: 0.20 g 100 L^−1^ lipase, *Mucor miehei*; L2: 0.40 g 100 L^−1^ lipase, *Mucor miehei*. ** PV: Penetrometer value; TA: Titratable acidity expressed as grams of lactic acid per 100 grams of cheese.

**Table 4:** Nitrogen fractions of experimental White cheeses during ripening (*n* = 3).

	Day	1	15	30	60	90
WSN ** (%)	C *	0.193 ± 0.003E ^c^	0.268 ± 0.005D ^b^	0.306 ± 0.004C ^c^	0.362 ± 0.009B ^b^	0.428 ± 0.013A ^b^
	**P1**	0.216 ± 0.005E ^ab^	0.288 ± 0.003D ^a^	0.329 ± 0.004C ^b^	0.404 ± 0.005B ^a^	0.462 ± 0.003A ^a^
	**P2**	0.229 ± 0.009E ^a^	0.289 ± 0.012D ^a^	0.350 ± 0.011C ^a^	0.391 ± 0.015B ^a^	0.451 ± 0.014A ^a^
	**L1**	0.196 ± 0.011D ^c^	0.268 ± 0.001C ^b^	0.304 ± 0.018B ^c^	0.395 ± 0.006A ^a^	0.393 ± 0.010A ^c^
	**L2**	0.209 ± 0.014E ^bc^	0.272 ± 0.001D ^b^	0.335 ± 0.003C ^ab^	0.364 ± 0.017B ^b^	0.405 ± 0.001A ^c^
**TCA-SN (%)**	**C**	0.154 ± 0.005E ^ab^	0.188 ± 0.013D ^a^	0.235 ± 0.003C ^bc^	0.269 ± 0.001B ^b^	0.306 ± 0.011A ^b^
	**P1**	0.156 ± 0.002E ^ab^	0.205 ± 0.009D ^a^	0.240 ± 0.005C ^bc^	0.295 ± 0.005B ^a^	0.339 ± 0.002A ^a^
	**P2**	0.164 ± 0.012E ^a^	0.202 ± 0.010D ^a^	0.255 ± 0.003C ^a^	0.293 ± 0.007B ^a^	0.323 ± 0.003A ^a^
	**L1**	0.141 ± 0.006D ^c^	0.184 ± 0.003C ^a^	0.227 ± 0.001B ^c^	0.299 ± 0.010A ^a^	0.294 ± 0.012A ^b^
	**L2**	0.151 ± 0.004D ^bc^	0.205 ± 0.012C ^a^	0.249 ± 0.017B ^ab^	0.267 ± 0.001B ^b^	0.306 ± 0.010A ^b^
**PTA-SN (%)**	**C**	0.051 ± 0.001E ^a^	0.054 ± 0.001D ^bc^	0.062 ± 0.001C ^a^	0.078 ± 0.002B ^bc^	0.083 ± 0.001A ^ab^
	**P1**	0.047 ± 0.001E ^b^	0.054 ± 0.001D ^bc^	0.064 ± 0.004C ^a^	0.080 ± 0.002B ^ab^	0.086 ± 0.002A ^a^
	**P2**	0.044 ± 0.001D ^c^	0.058 ± 0.002C ^a^	0.060 ± 0.002C ^a^	0.076 ± 0.001B ^c^	0.086 ± 0.001A ^a^
	**L1**	0.042 ± 0.001D ^d^	0.053 ± 0.001C ^cd^	0.056 ± 0.001B ^b^	0.081 ± 0.003A ^a^	0.080 ± 0.002A ^b^
	**L2**	0.046 ± 0.001C ^bc^	0.055 ± 0.001B ^b^	0.055 ± 0.001B ^b^	0.077 ± 0.002A ^c^	0.079 ± 0.004A ^b^
**CN (%)**	**C**	2.53 ± 0.050A ^a^	2.53 ± 0.021A ^a^	2.53 ± 0.098A ^b^	2.48 ± 0.061A ^a^	2.45 ± 0.023A ^ab^
	**P1**	2.50 ± 0.056A ^a^	2.36 ± 0.020C ^c^	2.55 ± 0.012A ^b^	2.45 ± 0.015B ^a^	2.38 ± 0.010C ^bc^
	**P2**	2.49 ± 0.021B ^a^	2.46 ± 0.036B ^ab^	2.69 ± 0.032A ^a^	2.48 ± 0.021B ^a^	2.33 ± 0.070C ^c^
	**L1**	2.55 ± 0.042A ^a^	2.36 ± 0.010B ^c^	2.56 ± 0.035A ^b^	2.59 ± 0.110A ^a^	2.41 ± 0.030B ^bc^
	**L2**	2.52 ± 0.036B ^a^	2.44 ± 0.087B ^bc^	2.68 ± 0.065A ^a^	2.51 ± 0.026B ^a^	2.53 ± 0.064B ^a^
**CN/TN (%)**	**C**	92.92 ± 0.050A ^a^	90.42 ± 0.121B ^a^	89.22 ± 0.474C ^a^	87.25 ± 0.065D ^a^	85.14 ± 0.471E ^b^
	**P1**	92.06 ± 0.321A ^bc^	89.13 ± 0.191B ^c^	88.57 ± 0.115C ^a^	85.85 ± 0.220D ^c^	83.74 ± 0.128E ^c^
	**P2**	91.59 ± 0.356A ^c^	89.47 ± 0.472B ^bc^	88.49 ± 0.311C ^a^	86.39 ± 0.525D ^bc^	83.76 ± 0.616E ^c^
	**L1**	92.86 ± 0.411A ^a^	89.79 ± 0.040B ^b^	89.38 ± 0.642B ^a^	86.78 ± 0.447C ^ab^	85.97 ± 0.375D ^a^
	**L2**	92.34 ± 0.578A ^ab^	89.98 ± 0.341B ^ab^	88.87 ± 0.321C ^a^	87.33 ± 0.624D ^a^	86.19 ± 0.250E ^a^
**PPN (%)**	**C**	0.038 ± 0.003C ^a^	0.081 ± 0.015B ^a^	0.071 ± 0.008B ^a^	0.094 ± 0.009B ^a^	0.123 ± 0.020A ^ab^
	**P1**	0.059 ± 0.003D ^a^	0.083 ± 0.006C ^a^	0.063 ± 0.006D ^a^	0.109 ± 0.006B ^a^	0.122 ± 0.002A ^ab^
	**P2**	0.065 ± 0.020C ^a^	0.088 ± 0.013BC ^a^	0.094 ± 0.009B ^a^	0.098 ± 0.017B ^a^	0.128 ± 0.010A ^a^
	**L1**	0.055 ± 0.010B ^a^	0.084 ± 0.004A ^a^	0.077 ± 0.019A ^a^	0.096 ± 0.009A ^a^	0.100 ± 0.014A ^b^
	**L2**	0.058 ± 0.015C ^a^	0.067 ± 0.011BC ^a^	0.087 ± 0.014AB ^a^	0.098 ± 0.016A ^a^	0.099 ± 0.009A^b^
**PPN/TN (%)**	**C**	1.40 ± 0.123C ^a^	2.89 ± 0.530B ^a^	2.50 ± 0.325B ^a^	3.29 ± 0.235B ^a^	4.25 ± 0.709A ^ab^
	**P1**	2.19 ± 0.146D ^a^	3.13 ± 0.244C ^a^	2.20 ± 0.195D ^a^	3.82 ± 0.214B ^a^	4.30 ± 0.081A ^ab^
	**P2**	2.39 ± 0.760E ^a^	3.18 ± 0.447C ^a^	3.10 ± 0.271D ^a^	3.42 ± 0.621B ^a^	4.60 ± 0.392A ^a^
	**L1**	2.00 ± 0.351C ^a^	3.21 ± 0.139AB ^a^	2.70 ± 0.649BC ^a^	3.20 ± 0.190AB ^a^	3.56 ± 0.517A ^bc^
	**L2**	2.14 ± 0.594B ^a^	2.48 ± 0.437AB ^a^	2.88 ± 0.471AB ^a^	3.40 ± 0.572A ^a^	3.36 ± 0.368A ^c^

A,B,C,D,E Means in the same row followed by different letters were significantly different (*p* < 0.05); ^a,b,c,d^ Means in the same column followed by different letters significantly different (*p* < 0.05); * Cheeses: C: Control without enzymes; P1; 0.05 g 100 L^−1^ protease, *Bacillus subtilis*; P2: 0.10 g 100 L^−1^ protease, *Bacillus subtilis*; L1: 0.20 g 100 L^−1^ lipase, *Mucor miehei*; L2: 0.40 g 100 L^−1^ lipase, *Mucor miehei*. ** WSN, water-soluble nitrogen; TCA-SN, 12% trichloroacetic acid-soluble nitrogen; PTA-SN, 5% phosphotungstic acid-soluble nitrogen; CN, Caseine nitrogen; PPN, proteose peptone nitrogen. WSN, TCA-SN or PTA-SN values were expressed as % of total nitrogen.

**Table 5:** Concentration of individual free amino acids (FAA) of Beyaz cheeses (*n* = 3) (mg 100 g^−1^).

Amino Acids	30 Days			90 Days		
	C *	P1	P2	C	P1	P2
Alanine (Ala)	59 ± 1 ^a^	60 ± 5 ^a^	72 ± 6 ^a^	70 ± 3 ^a^	76 ± 9 ^a^	75 ± 3 ^a^
Glycine (Gly)	38 ± 5 ^a^	37 ± 4 ^a^	44 ± 3 ^a^	42 ± 3 ^a^	42 ± 9 ^a^	42 ± 4 ^a^
Valine (Val)	137 ± 19 ^a^	128 ± 13 ^a^	143 ± 21 ^a^	150 ± 11 ^a^	182 ± 26 ^a^	176 ± 12 ^a^
Leucine (Leu)	224 ± 24 ^a^	219 ± 23 ^a^	250 ± 9 ^a^	245 ± 11 ^a^	282 ± 39 ^a^	271 ± 6 ^a^
Isoleucine (Ile)	116 ± 12 ^a^	98 ± 6 ^a^	108 ± 14 ^a^	122 ± 6 ^a^	149 ± 25 ^a^	145 ± 1 ^a^
Threonine (Thr)	78 ± 9 ^a^	85 ± 6 ^a^	97 ± 6 ^a^	89 ± 13 ^a^	102 ± 20 ^a^	95 ± 1 ^a^
Serine (Ser)	86 ± 17 ^a^	104 ± 8 ^a^	128 ± 12 ^a^	112 ± 4 ^a^	107 ± 10 ^a^	110 ± 2 ^a^
Proline (Pro)	217 ± 19 ^b^	238 ± 9 ^a^	252 ± 26 ^a^	234 ± 9 ^b^	269 ± 10 ^a^	273 ± 9 ^a^
Aspartate (Asp)	180 ± 40 ^a^	170 ± 6 ^a^	253 ± 29 ^b^	192 ± 13 ^b^	239 ± 49 ^b^	349 ± 28 ^a^
Methionine (Met)	29 ± 3 ^a^	29 ± 2 ^a^	25 ± 1 ^a^	29 ± 1 ^b^	51 ± 8 ^a^	55 ± 12 ^a^
Glutamate (Glu)	279 ± 11 ^b^	271 ± 22 ^b^	369 ± 32 ^a^	327 ± 21 ^b^	369 ± 27 ^ab^	374 ± 28 ^a^
Phenylalanine (Phe)	118 ± 13 ^a^	130 ± 8 ^a^	133 ± 5 ^a^	140 ± 9 ^a^	153 ± 27 ^a^	151 ± 1 ^a^
Lysine (Lys)	141 ± 12 ^a^	142 ± 10 ^a^	169 ± 15 ^a^	152 ± 8 ^a^	152 ± 9 ^a^	162 ± 2 ^a^
Histidine (His)	41 ± 2 ^a^	45 ± 2 ^a^	44 ± 6 ^a^	39 ± 0 ^c^	45 ± 2 ^b^	50 ± 2 ^a^
Tyrosine (Tyr)	101 ± 5 ^a^	100 ± 6 ^a^	107 ± 9 ^a^	104 ± 2 ^a^	111 ± 4 ^a^	115 ± 2 ^a^

^a,b,c^: Average values in the same row and days with different letters are significantly different (*p* < 0.05). * Cheeses: C: Control without enzymes; P1; 0.05 g 100 L^−1^ protease, *Bacillus subtilis*; P2: 0.10 g 100 L^−1^ protease, *Bacillus subtilis*; L1: 0.20 g 100 L^−1^ lipase, *Mucor miehei*; L2: 0.40 g 100 L^−1^ lipase, *Mucor miehei*.

**Table 6:** Mineral contents (mg 100g^−1^) of experimental White cheeses during ripening (*n* = 3).

Mineral	Day	C *	P1	P2	L1	L2
Ca	30	726 ± 01 ^ab^	687 ± 19 ^b^	699 ± 40 ^b^	723 ± 37 ^ab^	764 ± 17 ^a^
	90	669 ± 16 ^bc^	646 ± 33 ^cd^	631 ± 13 ^d^	698 ± 10 ^ab^	718 ± 18 ^a^
P	30	698 ± 11 ^a^	645 ± 09 ^a^	677 ± 16 ^a^	706 ± 18 ^a^	713 ± 08 ^a^
	90	671 ± 03 ^ab^	641 ± 05 ^c^	631 ± 12 ^bc^	646 ± 28 ^bc^	704 ± 10 ^a^
Na	30	1968 ± 02 ^a^	1728 ± 26 ^b^	1933 ± 40 ^a^	1928 ± 89 ^a^	1665 ± 14 ^b^
	90	1860 ± 25 ^a^	1560 ± 36 ^d^	1724 ± 83 ^b^	1704 ± 30 ^bc^	1612 ± 61 ^cd^
K	30	253 ± 05 ^ab^	253 ± 16 ^c^	231 ± 06 ^b^	233 ± 09 ^a^	226 ± 21 ^a^
	90	188 ± 17 ^b^	161 ± 04 ^c^	183 ± 10 ^c^	173 ± 13 ^bc^	209 ± 15 ^a^
Mg	30	210 ± 08 ^a^	211 ± 07 ^a^	204 ± 07 ^a^	209 ± 06 ^a^	209 ± 02 ^a^
	90	179 ± 03 ^a^	179 ± 09 ^a^	175 ± 04 ^a^	185 ± 07 ^a^	189 ± 05 ^a^
Zn	30	4.63 ± 0.070 ^a^	4.02 ± 0.030 ^d^	4.43 ± 0.060 ^b^	4.09 ± 0.069 ^d^	4.33 ± 0.023 ^c^
	90	3.95 ± 0.040 ^a^	4.00 ± 0.047 ^a^	3.80 ± 0.072 ^b^	3.83 ± 0.017 ^b^	4.00 ± 0.055 ^a^
Fe	30	0.407 ± 0.038 ^a^	0.389 ± 0.017 ^a^	0.385 ± 0.011 ^a^	0.391 ± 0.002 ^a^	0.365 ± 0.028 ^a^
	90	0.379 ± 0.015 ^ab^	0.361 ± 0.017 ^b^	0.393 ± 0.009 ^a^	0.390 ± 0.015 ^a^	0.363 ± 0.004 ^b^
Cu	30	0.107 ± 0.011 ^a^	0.065 ± 0.005 ^b^	0.070 ± 0.009 ^b^	0.078 ± 0.014 ^b^	0.073 ± 0.001 ^b^
	90	0.110 ± 0.001 ^a^	0.067 ± 0.002 ^b^	0.069 ± 0.007 ^b^	0.071 ± 0.010 ^b^	0.069 ± 0.005 ^b^
Mn	30	0.058 ± 0.001 ^a^	0.056 ± 0.005 ^a^	0.055 ± 0.004 ^a^	0.055 ± 0.001 ^a^	0.057 ± 0.002 ^a^
	90	0.053 ± 0.006 ^a^	0.045 ± 0.002 ^a^	0.047 ± 0.000 ^a^	0.044 ± 0.004 ^a^	0.048 ± 0.001 ^a^

^a,b,c,d^ Means in the same row followed by different letters were significantly different (*p* < 0.05); * Cheeses: C: Control without enzymes; P1; 0.05 g 100 L^−1^ protease, *Bacillus subtilis*; P2: 0.10 g 100 L^−1^ protease, *Bacillus subtilis*; L1: 0.20 g 100 L^−1^ lipase, *Mucor miehei*; L2: 0.40 g 100 L^−1^ lipase, *Mucor miehei*.
